# How similar are the molecular mechanisms of yeast and metazoan genome replication initiation?

**DOI:** 10.1042/BST20220917

**Published:** 2025-03-07

**Authors:** Giacomo Palm, Alessandro Costa

**Affiliations:** Macromolecular Machines Laboratory, The Francis Crick Institute, London NW1 1AT, U.K

**Keywords:** DONSON, MCM, origin licensing, replication fork

## Abstract

DNA replication start sites are licensed for replication when two hexameric ring-shaped motors of the replicative helicase are loaded as an inactive double hexamer around duplex DNA. Activation requires untwisting of the double helix and ejection of one DNA strand from the central channel of each helicase ring. The process of replication initiation is best understood in yeast, thanks to reconstitution with purified yeast proteins, which allowed systematic structural analysis of the replication initiation process. Orthologs of most yeast replication factors have been identified in higher eukaryotes; however, reconstitution of metazoan replication initiation is still in its infancy, with double hexamer loading but not activation having been achieved. Nonetheless, artificial intelligence-driven structure prediction and cryo-EM studies on native complexes, combined with cell-based and cell-free approaches, are starting to provide insights into metazoan replication initiation mechanisms. Here, we describe the emerging picture.

## Introduction

Any DNA segment in our chromosomes must be duplicated once and only once per cell cycle to maintain genome stability. To achieve this, eukaryotic cells have evolved to temporally separate loading and activation of the helicase enzyme that unwinds DNA for replication [1,2]. During late mitosis and throughout the G1 phase of the cell cycle, the hetero-hexameric Mcm2-7 [hereafter minichromosome maintenance (MCM)] motor of the replicative helicase is loaded onto DNA as a head-to-head double hexamer (DH) that contains the symmetry to support bidirectional replication but remains catalytically inactive [3,4]. Activation occurs upon S phase transition, when two firing factors, cell division cycle protein 45 (Cdc45) and Go-Ichi-Ni-San (GINS), are recruited to MCM, together forming the Cdc45-MCM-GINS (CMG) holohelicase [5–7]. The same cyclin-dependent kinase (CDK) phosphorylation signal that triggers activation also prevents loading of MCM DHs onto newly synthesised DNA [8], achieving once-per-cell-cycle replication. CMG is the organising centre of the DNA unwinding and synthesis machinery at the replication fork, and its assembly requires a cascade of sequential events [7,9]. How to build a replisome is best understood in yeast, thanks to more than 30 years of genetic and biochemical studies [10], which culminated in the reconstitution of replication initiation in a test tube with purified yeast proteins [9]. Biochemical staging of initiation, in turn, allowed for the cryo-EM [11–18] and single-molecule visualisation of this process [19–25], which started to explain the physical mechanisms of the activation of replication start sites (origins). Given that orthologs for most factors recognised to be essential for replication have been identified in metazoans, findings obtained with yeast are generally deemed useful to inform eukaryotic replication processes. Examples such as replisome architecture [26–29] and translocation mechanism of the CMG helicase [26,30,31] indicate that this assumption is largely valid. However, recent findings from human [32–34], frog egg extracts and the worm systems [35–39] have revealed unexpected differences in the complement of replisome assembly factors, and in the physical mechanisms enabling activation of metazoan origins, compared with yeast. In this article, we review recent progress in our understanding of replication initiation across eukaryotic species.

### Origin licensing reconstituted with yeast proteins

**Figure 1:**
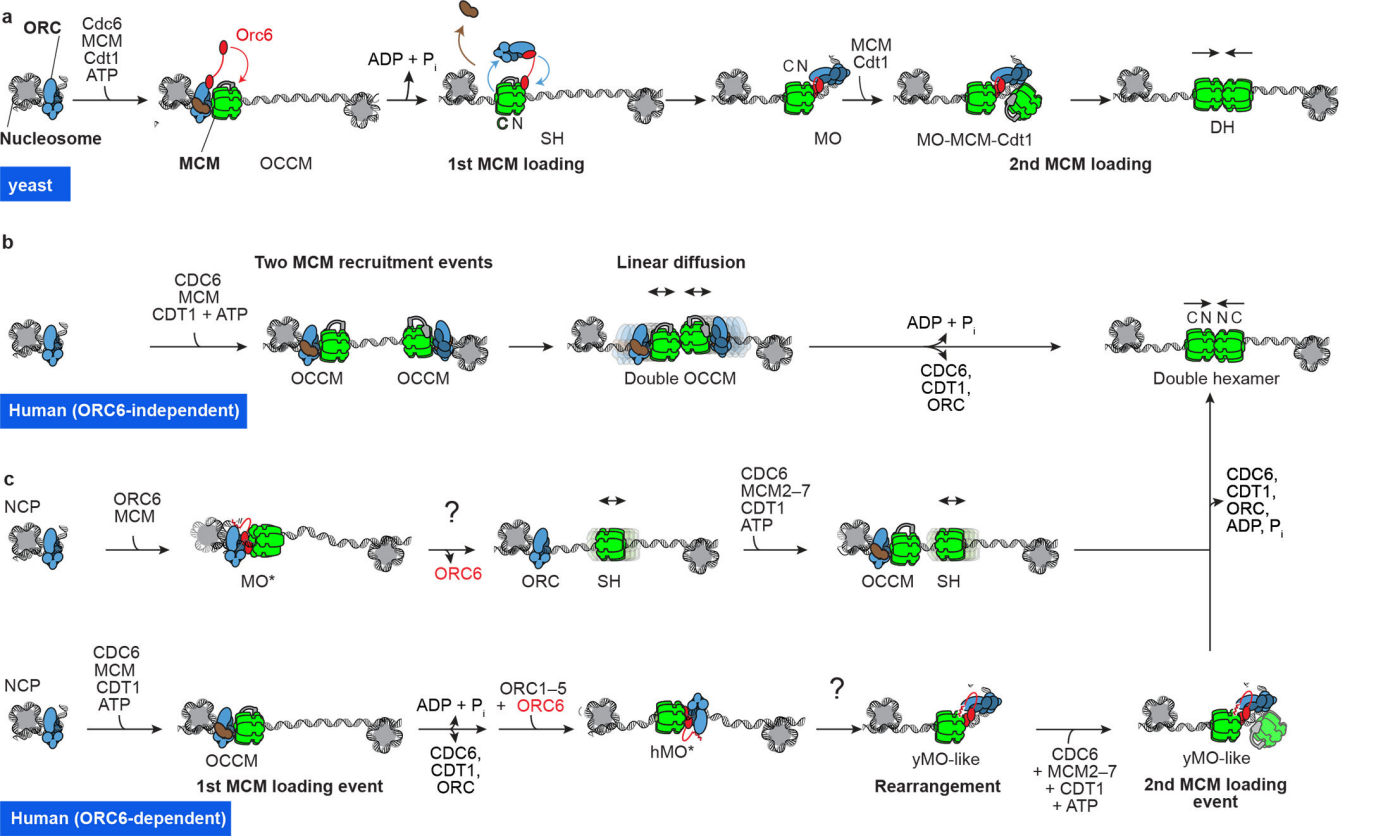
Origin licensing mechanisms elucidated by cryo-EM. (**A**) Yeast DH loading. (**B**) Human DH loading in the absence of ORC6 relies on two MCM (or OCCM) complexes passively diffusing along duplex DNA and engaging their N-terminal domains. (**C**) Two possible mechanisms for human DH loading dependent on ORC6. ORC6 could engage ORC1-5 and recruit an SH by engaging its N-terminal side. SH release followed by OCCM formation would result in two SHs with orientation competent for forming a DH. Alternatively, OCCM could form, followed by MCM departure from ORC. ORC6 would then bind N-terminal MCM resulting in a closed MO configuration (marked as MO*), incompetent for second MCM recruitment. A conformational change might occur which opens the MO structure, visiting the conformer observed with yeast proteins. This yeast-like MO state would be competent for second hexamer recruitment via the OCCM pathway, eventually leading to DH formation. MCM, minichromosome maintenance; OCCM, ORC-Cdc6-Cdt1-MCM; ORC; origin recognition complex; SH, single MCM hexamer.

Reconstitution of MCM loading onto a yeast origin of replication established that this process is strictly sequential. First, the origin recognition complex (ORC) recognises a high-affinity site named EACS/B1. ORC is a six-membered ATPase complex that binds [40] and bends [41] DNA and recruits a separate ATPase subunit [cell division cycle protein 6 (Cdc6)] [42]. Cdc6 engages ORC, in turn closing a ring around duplex DNA [43–45]. The C-terminal side of the ORC-Cdc6 ring serves as a landing platform for the recruitment of MCM-Cdt1 [46]. This is a loading-competent form of the helicase motor that contains a discontinuity between the Mcm2 and Mcm5 subunits [3,47,48], which serves as a gate for DNA entry [49]. ATP binding by ORC-Cdc6 and MCM-Cdt1 achieves threading of duplex DNA inside the MCM central pore, leading to the assembly of a helicase loading intermediate (ORC-Cdc6-Cdt1-MCM, or OCCM), where the ORC-Cdc6 and MCM-Cdt1 rings align and interact via their C-termini [50–52]. ATP hydrolysis by MCM achieves the disengagement from ORC-Cdc6 and the ejection of Cdt1 [53,54], leading to the loading of a single MCM hexamer (SH) [55]. Duplex DNA within the SH pore is engaged by a set of ATPase pore loops that primarily track along the leading strand [55]. ATP hydrolysis at the Orc1/Cdc6 ATPase site ejects Cdc6 and allows ORC to flip from the C- to the N-terminal side of the single-loaded MCM and engage a lower affinity DNA element named B2 [13]. In this configuration, Orc6 bridges between MCM and ORC, straddling across the closed Mcm2-5 gate and forming an MO complex that is competent for the recruitment of a second MCM via the same OCCM mechanism as the first. This will eventually achieve DH loading, when the N-termini from two hexamers engage [56] (Figure 1a). In the DH, the ATPase pore loops rearrange to engage in an equal number of contacts with both leading and lagging strands. The two MCM hexamers are slightly misaligned, so that duplex DNA bends as it traverses the central pore of the DH. Despite this distortion, the double helix maintains a B-form character [11,12]. Phosphorylation by S-CDK breaks the DH loading process, by targeting two ORC subunits, which interferes with both OCCM and MO formation [55,57,58]. When MO assembly is impaired, DH formation can still occur through the independent recruitment of two SHs that meet, probably through random diffusion along duplex DNA [55].

### Origin licensing reconstituted with human proteins

Although human MCMs are loaded by orthologs of yeast ORC, Cdc6 and Cdt1, several differences in the mechanism have been uncovered over the years. First, human ORC lacks the structural elements conferring specificity to origin-DNA binding in yeast [41,59–61]. Second, the DNA entrapped in human DHs purified from cells is untwisted and melted at the N-terminal dimerisation interface. Two Mcm5 residues, Arg195 and Leu209, symmetrically stabilise two unpaired bases [32–34]. Arg195 is conserved throughout eukaryotic Mcm5s, but the corresponding yeast residue (Arg209) has not been observed engaged in DNA opening in any of the MCM-containing structures characterised so far. Reconstitution of DH formation with purified human proteins established that loading alone is sufficient to nucleate DNA melting, indicating that this is a feature specific to human MCM loading and does not reflect a downstream activation intermediate yet to be discovered in yeast [33,34]. A third difference is the process of MCM loading itself, which departs from the stringently sequential cascade of events enabling yeast DH formation. For example, unlike in yeast, CDC6 recruitment by ORC stimulates but is not required for human MCM loading [34]. The structural intermediates obtained through cryo-EM imaging of the complete loading reaction support this biochemical observation. In fact, not only ORC-Cdc6 but also ORC alone can be captured in the act of recruiting MCM-Cdt1 onto origin DNA [33]. The role of ORC6 is also different from yeast, as it stimulates MCM loading by wildtype ORC, but it is not essential for DH formation in a test tube or for viability in cells [62]. Indeed, it inhibits loading when an ORC variant is used, which lacks a metazoan-specific N-terminal ORC1 domain containing an intrinsically disordered region [33]. While the structural mechanism is not understood, a suggestive nucleoprotein assembly (human MO*) has been observed when imaging human DH loading using the truncated ORC1 variant. Like in yeast MO, human ORC engages N-terminal MCM via ORC6; however, it visits a closed configuration that could not possibly support DH formation. In fact, ORC6 blocks the homo-dimerisation interface and the orientation of the ORC1-5 loading platform is such that recruitment of a second MCM hexamer cannot occur due to steric clashes [33]. Negative stain analysis of DH loading performed by the Bleichert group, using an internal truncation in N-terminal ORC1 (Δ400–861), indicates that a yeast-like human MO structure can be assembled [34]. What favours the formation of either structure and whether a regulatory mechanism exists, which might drive conversion between the MO* and MO states, remains to be established. Finally, unlike for yeast, human SH loading and MO* assembly do not require Cdt1, nor ATP hydrolysis by MCM. This suggests that recruitment of a single helicase might occur via direct engagement of N-terminal MCM by ORC. At least one of two MCM hexamers, however, must be recruited through C-terminal engagement (i.e. the OCCM pathway) for DH loading to be completed [34]. The emerging picture indicates that multiple pathways exist, ORC6 dependent and independent, which lead to the engagement of two human SHs to form a DH (Figure 1b–c).

### MCM helicase activation reconstituted with yeast proteins

**Figure 2:**
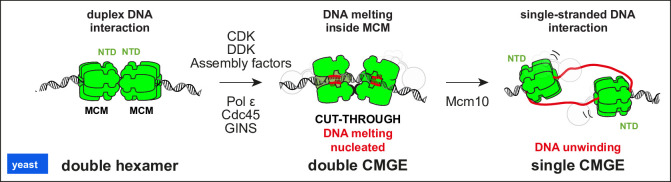
Stepwise separation of two MCM hexamers leads to origin DNA opening. DDK and CDK phosphorylations facilitate the recruitment of assembly factors that deposit Cdc45, GINS and Pol epsilon onto MCM. This leads to the partial separation of the two MCM rings and untwisting of DNA within the central pore of the helicase, through the interaction of ATPase elements. Mcm10 recruitment leads to ejection of the lagging strand template and activation of the single-stranded DNA translocation function of MCM, which leads the two helicases to cross paths and establish two divergent replication forks. CDK, cyclin-dependent kinase; Cdc45, cell division cycle protein 45; DDK, Dbf4-dependent kinase; GINS, Go-Ichi-Ni-San; MCM, minichromosome maintenance.

DH phosphorylation by the Dbf4-dependent kinase (DDK, aka Cdc7-Dbf4) is the first step towards the activation of the DNA unwinding function of the MCM ATPase motor [63]. N-terminal Dbf4 engages the Mcm2 domain of one MCM hexamer placing Cdc7 in a suitable position to phosphorylate N-terminal Mcm4 on the opposed hexamer, effectively recognising the three-dimensional shape of a loaded DH [18]. In the process of docking onto Mcm4, C-terminal Dbf4 evicts from a self-inhibitory docking site the N-terminal tail of Mcm4, which contains the DDK phosphorylation targets. Phosphorylated N-terminal Mcm4 no longer engages this site after DDK release, exposing a new MCM epitope that might be recognised by one of the CMG assembly factors [14,15]. A candidate assembly factor is Sld3/7, which is known to bind the phosphorylated DH and deliver Cdc45 onto MCM, forming a transient complex [64]. GINS recruitment to MCM-Cdc45 is understood to require Dpb11, Sld2, Pol epsilon assembly factors and S-CDK, which have been observed to form a so-called pre-loading complex with GINS [65–67]. Although the process that completes CMG formation is not understood at the molecular level, it is known that the CDK kinase targets Sld2 and Sld3, which are, in turn, recognised by the Dpb11 phospho-reader [67,68]. Cdc45 and GINS latch across the N-terminal side of Mcm2-5-3 [69], and Pol epsilon engages the same MCM subunits on the C-terminal side [28]. CMG formation leads to the partial separation of two CMGE assemblies, which remain tethered via Mcm6 alone. Within this double CMGE complex, a change in the ATPase pore loops engaging duplex DNA leads to local untwisting of DNA, with widening of the minor groove and nucleation of DNA melting. An Mcm6-specific element that mediates homo-dimerisation in the DH invades the MCM central channel of the dCMGE and stabilises the orphan bases created upon DNA melting by the MCM ATPase. A duplex DNA segment entrapped between the two MCM hexamers of the double CMGE maintains a B-form character [16]. The events leading to full untwisting of duplex DNA inside the double CMGE still need to be described. It is known, however, that Mcm10 recruitment leads to extensive ATP-dependent DNA unwinding by the CMG and RPA recruitment [7] (compatible with other work implicating Mcm10 in origin activation [70–72]). Cryo-EM analysis showed that DNA unwinding is coupled with the separation of two CMGE assemblies. At the same time, a change in the structure of the ATPase ring promotes selective ejection of the lagging strand from the MCM central channel [17]. While the identity of the MCM subunit interface that serves as DNA escape gate remains to be discovered, it is known that two CMGEs cross paths as they establish two divergent replication forks (Figure 2) [7,30,73].

### MCM helicase activation in metazoans

Our understanding of origin activation in metazoans derives from cellular and biochemical analysis, mainly with the mammalian [74], worm [38] and frog egg extract systems [35–37,39,75]. Furthermore, although the process of CMG assembly is yet to be reconstituted with purified proteins, artificial intelligence-driven structure prediction [76] and cryo-EM analysis [38,77] on native initiation complexes [36], along with cell biology and cell-free studies, started to provide a glimpse of the molecular mechanism.

**Figure 3:**
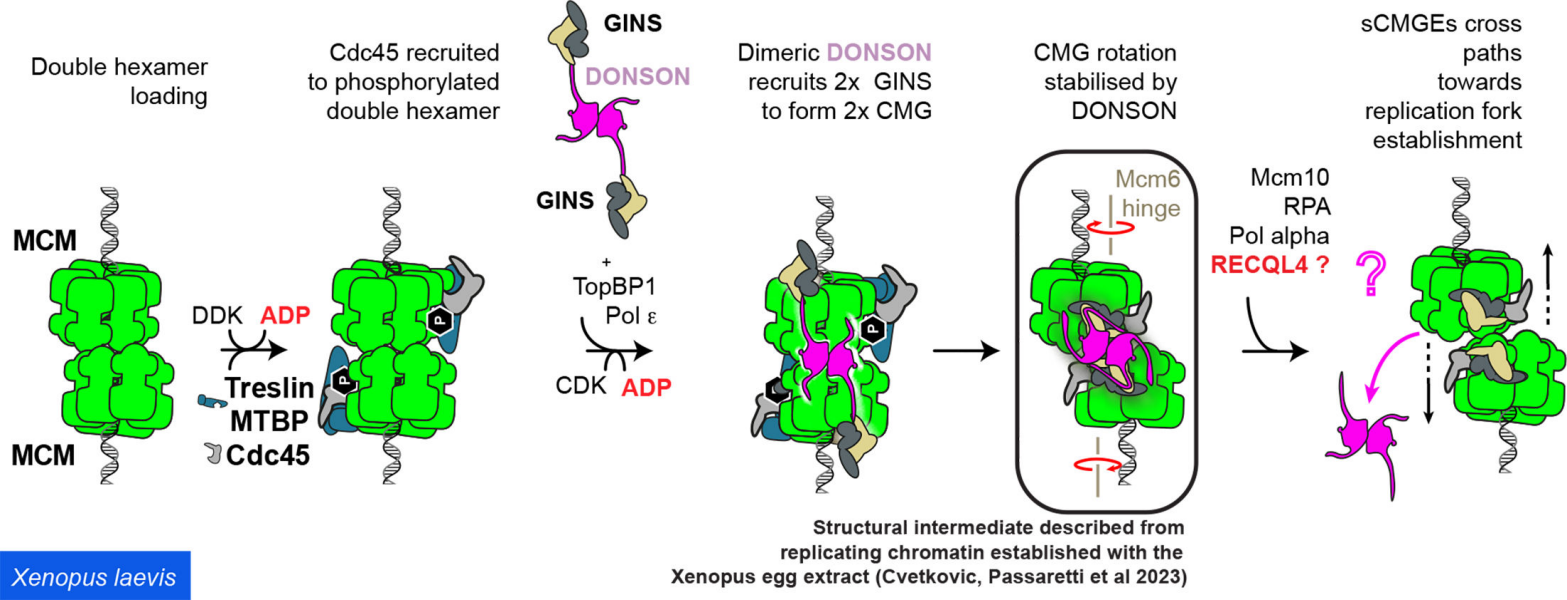
Origin activation in metazoans. TRESLIN-MTBP recruit Cdc45. A DONSON dimer recruits two copies of GINS to MCM in a poorly understood process that also involves TOPBP1 and Pol epsilon. DONSON engagement of MCM also changes the structure of the DH that becomes misaligned, in a manner that likely causes DNA melting at the MCM dimerisation interface. RECQL4 promotes DONSON eviction in a poorly understood process, which, together with MCM10, leads to the separation of two CMG assemblies and establishment of divergent replication forks, with leading and lagging strand synthesis primed by Pol alpha. Cdc45, cell division cycle protein 45; CMG, Cdc45-MCM-GINS; DONSON, downstream neighbor of son; GINS, Go-Ichi-Ni-San; MCM, minichromosome maintenance.

Orthologs of most factors known to be involved in CMG assembly in yeast have been identified in metazoans. These include TRESLIN [78,79] (yeast Sld3), MTBP [80] (Sld7) and TOPBP1 [81] (Dpb11). The identification of metazoan Sld2 has been more contentious. While an ortholog of Sld2 has been identified in *Caenorhabditis elegans [82],* most metazoans contain RecQL4, a helicase enzyme featuring an Sld2 homology domain at the N-terminus. Unlike the yeast-based consensus model, which implicates Sld2 in GINS recruitment to MCM, RecQL4 acts during an ill-defined origin activation step that occurs downstream of CMG formation [83,84]. Instead, the GINS recruitment function is provided by downstream neighbor of son (DONSON), a CMG-assembly factor, which is not present in yeast [35,37–39,74]. An initiation intermediate, purified from replicating chromatin established with the *Xenopus* egg extract, revealed that DONSON assembles as a homodimer that engages two MCM3 subunits, and symmetrically recruits two copies of GINS [36]. The exact same observations were made through structure prediction using AlphaFold-multimer [37], and through the reconstitution of recombinant worm CMG replisome bound to DONSON [38]. Only the structure of the native initiation intermediate from the frog extract, however, provided a complete view of the DONSON-bound initiation intermediate, revealing that DONSON engagement also reconfigures the MCM DH [36]. To render MCM3 binding possible across the DH, in fact, DONSON changes the relative orientation of the two hexamers so that the central channels become misaligned. Although the endogenous double CMG–DONSON complex was purified after chromatin digestion, resulting in a DNA-free structure, the orientation of the two helicase motors appears incompatible with B-form duplex DNA traversing the narrow lumen at the N-terminal dimerisation interface of MCM [36]. Thus, it appears that DONSON not only delivers GINS to MCM but also changes the structure of the double CMG in a fashion that would promote untwisting of the double helix (Figure 3). Coherent with the notion that DONSON acts during origin-DNA opening is the observation that RNAi depletion of DONSON is synthetic lethal with a near-complete Mcm10 deletion in *C. elegans* [38].

A puzzling observation remains. The architecture of multiple MCM activation intermediates has been described using cryo-EM imaging of the reconstituted origin-dependent system with yeast proteins. However, never has a yeast structure been observed which resembles the distorted metazoan double CMG stabilised by DONSON. It will be important to establish whether this reflects a completely new origin unwinding mechanism that does not exist in yeast, or rather deeper structural analysis of *Saccharomyces cerevisiae* origin activation will uncover a CMG-DONSON-like intermediate important for activation.

Future questions also pertain to downstream origin activation events. Structural work, in fact, revealed that Pol alpha engages the CMG on the same MCM3 site occupied by DONSON [27]. This observation indicates that DONSON must be released as the double CMG is broken to establish bidirectional DNA replication [36–38]. While this process is still not understood at the molecular level, single-molecule fluorescence work on origin activation (established with the Xenopus egg extract) revealed that RecQL4 recruitment leads to the release of DONSON and triggers bidirectional replication [75]. The observation that a RecQL4 knockout in human cells is viable indicates that some redundancy likely exists in the mechanism underlying the establishment of two divergent replication forks [85]. Further investigation, including reconstitution in a test tube with purified proteins, will be needed to reach a molecular understanding of metazoan origin activation. In this respect, it remains unknown whether any metazoan-specific factors essential for origin activation remain to be identified.

**Perspectives**

- Replication initiation is highly regulated to ensure that replication occurs only once per cell cycle.
- Most of the factors important for replication fork establishment in yeast are found in metazoans. However, yeast does not contain DONSON, the factor that delivers GINS to MCM and changes how two MCM rings are aligned on the path to separation and establishment of two divergent replication forks. Does that mean that yeast and metazoans use different mechanisms for opening origin DNA?
- Comparative structural work on origin DNA opening, the role of ATP hydrolysis by MCM and downstream activation factors such as MCM10 and RECQL4 will help gain a more complete understanding of origin firing.

## Abbreviations

CMG: Cdc45-MCM-GINS
Cdc6: cell division cycle 6
DONSON: downstream neighbor of son
GINS: Go-Ichi-Ni-San
MCM: minichromosome maintenance
OCCM: ORC-Cdc6-Cdt1-MCM
ORC: origin recognition complex
